# Driver Monitoring System Using Computer Vision for Real-Time Detection of Fatigue, Distraction and Emotion via Facial Landmarks and Deep Learning

**DOI:** 10.3390/s26030889

**Published:** 2026-01-29

**Authors:** Tamia Zambrano, Luis Arias, Edgar Haro, Victor Santos, María Trujillo-Guerrero

**Affiliations:** Departamento de Automatización y Control Industrial, Escuela Politécnica Nacional, Quito 170525, Ecuador; luis.arias02@epn.edu.ec (L.A.); edgar.haro01@epn.edu.ec (E.H.); victor.santos@epn.edu.ec (V.S.); maria.trujillo01@epn.edu.ec (M.T.-G.)

**Keywords:** driver monitoring system, drowsiness detection, distraction detection, emotion recognition, Eye Aspect Ratio (EAR), Mouth Aspect Ratio (MAR), deep learning

## Abstract

**Highlights:**

**What are the main findings?**

- The system detects drowsiness using the EAR and the MAR with high accuracy (88.89% and 85.19%, respectively).
- Distraction behaviors based on head pose and gaze are detected with 100% accuracy.
- Emotion recognition performs well for happiness (100%), anger/disgust (96.3%), and surprise (92.6%), while the detection of sadness (66.7%) and fear (0%) remains more challenging due to atypical real-world expressions.
- The system operates in real time using a convolutional neural network based on MobileNetV2 and facial landmarks from MediaPipe.

**What are the implications of the main findings?**

- The real-time detection of fatigue and distraction can improve road safety and prevent accidents.
- Including emotion recognition enhances detection accuracy by providing context to facial cues.
- The use of non-invasive, efficient methods allows deployment in real driving environments.
- Identifying emotional and attentional states can support the development of smarter, adaptive vehicle safety systems.

**Abstract:**

Car accidents remain a leading cause of death worldwide, with drowsiness and distraction accounting for roughly 25% of fatal crashes in Ecuador. This study presents a real-time driver monitoring system that uses computer vision and deep learning to detect fatigue, distraction, and emotions from facial expressions. It combines a MobileNetV2-based CNN trained on RAF-DB for emotion recognition and MediaPipe’s 468 facial landmarks to compute the EAR (Eye Aspect Ratio), the MAR (Mouth Aspect Ratio), the gaze, and the head pose. Tests with 27 participants in both real and simulated driving environments showed strong results. There was a 100% accuracy in detecting distraction, 85.19% for yawning, and 88.89% for eye closure. The system also effectively recognized happiness (100%) and anger/disgust (96.3%). However, it struggled with sadness and failed to detect fear, likely due to the subtlety of real-world expressions and limitations in the training dataset. Despite these challenges, the results highlight the importance of integrating emotional awareness into driver monitoring systems, which helps reduce false alarms and improve response accuracy. This work supports the development of lightweight, non-invasive technologies that enhance driving safety through intelligent behavior analysis.

## 1. Introduction

Each year, more than 1.19 million people die due to car accidents, making them a major worldwide concern. In Ecuador, the situation is highly alarming. In 2024, the Agencia Nacional de Tránsito (ANT) reported that a person dies in a traffic accident every 3.8 h, with a total of 2302 fatalities and 18,312 injuries resulting from 21,220 accidents. This means that, on average, someone is injured every 30 min. According to the report, driver fatigue and inattention accounted for 25% of all incidents, more than other significant factors, such as speeding (15%) and traffic infractions (19%). These statistics reflected the critical need for intelligent systems that can monitor drivers to detect distractions and fatigue before they lead to fatal consequences [1].

Researchers have advanced the development of systems to monitor driver fatigue, distraction, and emotions in response to those alarming numbers. Fatigue detection has been widely explored through facial landmark-based approaches, particularly those employing metrics such as the Eye Aspect Ratio (EAR) and the Mouth Aspect Ratio (MAR). These indicators are used to assess prolonged eye closure and yawning, key signs of drowsiness. Prasanthi et al. implemented a real-time system based on the EAR and the MAR using OpenCV and Dlib, triggering alerts when thresholds are crossed [2]. Similarly, You et al. presented a deep learning-based system that addresses interindividual variability by combining CNNs and SVMs to personalize fatigue classification through eye morphology analysis, resulting in 94.80% accuracy [3]. These systems still experience difficulty with natural facial variations and outside conditions, but they show effectiveness in controlled settings.

In addition to traditional landmark-based techniques, Safarov et al. proposed a CNN-powered framework capable of the real-time detection of blinking and yawning, achieving 95.8% accuracy for eye-based fatigue [4]. By integrating facial landmark tracking with deep learning, the system enhanced robustness but did not address emotional expression interference. Their work highlights the effectiveness of combining deep learning and computer vision to detect drowsiness through eye and mouth movement patterns, leveraging real-time landmark-based analysis. Alguindigue et al. introduced an advanced approach to fatigue detection by integrating physiological parameters, notably heart rate variability (HRV) and electrodermal activity (EDA), into a deep neural network-based classification framework. Their findings demonstrated that the HRV-based model achieved precision and recall of 98%, indicating high accuracy. Nevertheless, the practicality of this method for real-time implementation in consumer vehicle settings remains limited due to its intrusive nature and operational complexity [5].

Other recent developments in distraction detection emphasize techniques such as analyzing head orientation and gaze deviations. A study by Schwarz et al. found that gaze and head movement analysis is effective for real-time driver state monitoring. Their Driver Monitoring System outperformed vehicle-based signals and reached its highest accuracy (AUC 0.897) when combined with vehicle data. However, it struggled to differentiate between moderate and severe drowsiness, indicating the need for additional inputs like time-of-day or physiological cues. As vehicle automation grows, traditional metrics lose relevance, making facial-based monitoring increasingly important [6]. Expanding on this, Díaz-Santos et al. implemented a system that attached facial recognition with continuous eye-tracking on edge devices to identify signs of drowsiness. While utilizing real-time image analysis and IoT technologies, their solution was effective for fatigue monitoring, but it still overlooked the role of emotional states [7].

Traditionally, emotion recognition has been treated independently from the analysis of fatigue or distraction. Beles et al. highlighted the advantages of employing multi-modal strategies by integrating facial expression analysis, ocular activity, and physiological signals to detect drowsiness in semi-autonomous driving contexts [8]. Nonetheless, their framework did not incorporate explicit emotion classification to enhance detection accuracy. In contrast, the present study introduces a novel approach that utilizes MobileNetV2, trained on the RAF-DB dataset, to classify emotional states and incorporate them as contextual modifiers in the detection pipeline. This methodology helps minimize false alarms by differentiating between fatigue-induced eye closure and momentary squinting resulting from expressions such as laughter or smiling, which are often misinterpreted through conventional systems.

Identifying the optimal strategy for real-world deployment remains a point of discussion within the field. Although physiological techniques demonstrate high accuracy, they are not well suited for non-invasive, in-vehicle applications. On the other hand, computer vision-based solutions offer greater scalability but are often compromised due to environmental and contextual interference. A key limitation of current systems is their failure to integrate emotional awareness into the logic of fatigue and distraction detection. This highlights the demand for comprehensive, real-time, and context-sensitive approaches. The proposed system addresses this challenge by integrating facial landmark detection, gaze and head pose estimation, and CNN-based emotion recognition into a unified framework. The analysis of facial cues in this study is limited to observable facial expressions suitable for real-time driving scenarios, rather than formal micro-expression analysis. Designed for real-time operation, the system includes an intelligent logging mechanism that selectively records significant behavioral changes, balancing technical precision with practical effectiveness to enhance driver safety.

## 2. Materials and Methods

**Figure 1:**
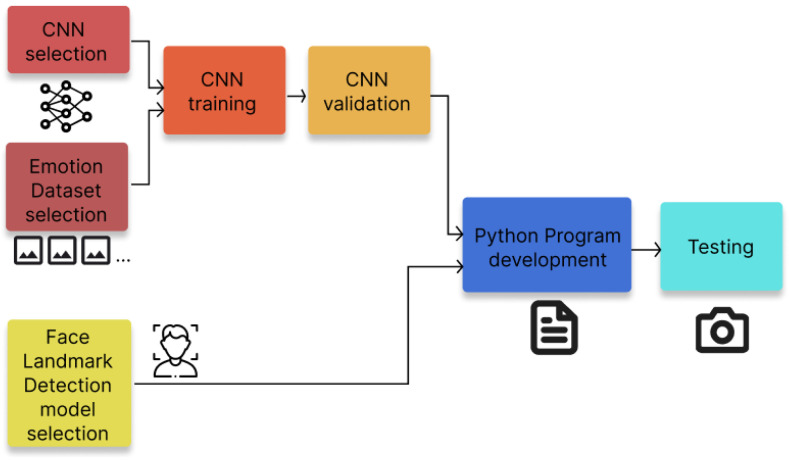
Design process of the developed system.

The system was developed in a Python 3.10 script that provides the drowsiness, distraction and emotion detection using the real-time video capture of a subject. To implement all of these detection functionalities, two major modules were included in the system design: a trained CNN for emotion detection and a pre-trained face landmark detection model necessary for the calculations of drowsiness and distraction parameters. Figure 1 summarizes the design process of the system.

### 2.1. Video Acquisition Setup

Real-time video data were acquired using a generic USB CMOS camera module compatible with the USB Video Class (UVC) standard. The camera module was manufactured by IMC Networks (Vendor ID: 13D3) and connected via a USB 2.0 interface. Video streams were captured at a spatial resolution of 640 × 480 pixels (VGA) with a frame rate of 30 frames per second. The camera was operated using the default UVC driver provided via the operating system, ensuring broad compatibility and reproducibility. These acquisition parameters were fixed for all experiments to allow consistent data capture and facilitate replication. Because the camera is compliant with the USB Video Class (UVC) standard, the acquisition setup can be replicated using any equivalent USB camera operating at the same resolution and frame rate, ensuring reproducibility across platforms.

### 2.2. Study Population

The experimental evaluation was conducted with a group of adult drivers representing a homogeneous cultural background and a range of driving experience levels. A summary of the main demographic and driving-related characteristics is provided in Table 1. These details are reported to contextualize the experimental results and clarify the scope of the conclusions drawn from this study.

### 2.3. Convolutional Neural Network for Emotion Detection

This study presents an emotion recognition system that employs a convolutional neural network based on MobileNetV2 [9]. It was chosen because of its efficiency in real-time applications. In order to identify seven different emotions—neutral, happy, sad, disturbed, angry, afraid, and surprised—the last layer was altered. Starting with pre-trained ImageNet weights, the model was fine-tuned using the RAF-DB dataset [10], which comprises roughly 30,000 labeled facial images representing a range of demographic groups and displaying different expressions under various lighting conditions. Additionally, a second dataset was used to test the program before the real-time tests. The second dataset is the “Driver Inattention Detection Dataset” [11], which contains almost 12,000 images of drivers in grayscale with 6 classes, dangerous driving, distracted driving, drinking, safe driving, sleepy driving and yawning; but just a small part of 2900 images with the classes, distracted driving, sleepy driving and yawning were considered for the test previously mentioned.

As part of the training process for the CNN, all images were resized to 224 × 224 pixels and normalized using standard ImageNet procedures. In an effort to enhance model robustness and improve generalization, some data augmentation techniques, such as brightness adjustments and random horizontal flipping, were implemented. The training process consisted of 15 epochs with a batch size of 32 samples per iteration.

In this training setup, cross-entropy loss was used as the objective function to measure how closely the model’s predictions matched the correct answers. This loss function involves higher penalties when the model is very confident but still makes a wrong prediction, which helps it learn to be both accurate and careful. For instance, if the model strongly predicts “happy” when the correct answer is “sad,” the loss will be much greater than if the model had been unsure. This method works especially well in emotion recognition tasks, as it helps the model better tell apart similar emotional expressions across different categories.

To optimize the model, the Adam optimizer was applied with a conservative learning rate of 0.0003. This relatively low rate encourages gradual and stable learning, often resulting in better overall performance, even if it extends the training time. Adam functions like an adaptive guide, adjusting the network’s internal parameters by taking into account both recent updates and longer-term patterns. It keeps track of past changes, allowing it to fine-tune how much each part of the model should adjust during training. By combining responsiveness with stability, Adam offers an effective strategy for navigating the complex optimization process.

After the training process was completed, the finalized model was saved and integrated into a real-time inference system. This integration made it possible to apply the model’s emotion recognition capabilities in practical, real-world contexts where the immediate analysis of emotional states was essential. During live operation, the system successfully analyzed facial expressions and classified them into predefined emotional categories, demonstrating consistent and reliable performance in real-time applications.

### 2.4. System for Drowsiness Detection Based on Eye Aspect Ratio (EAR)

**Figure 2:**
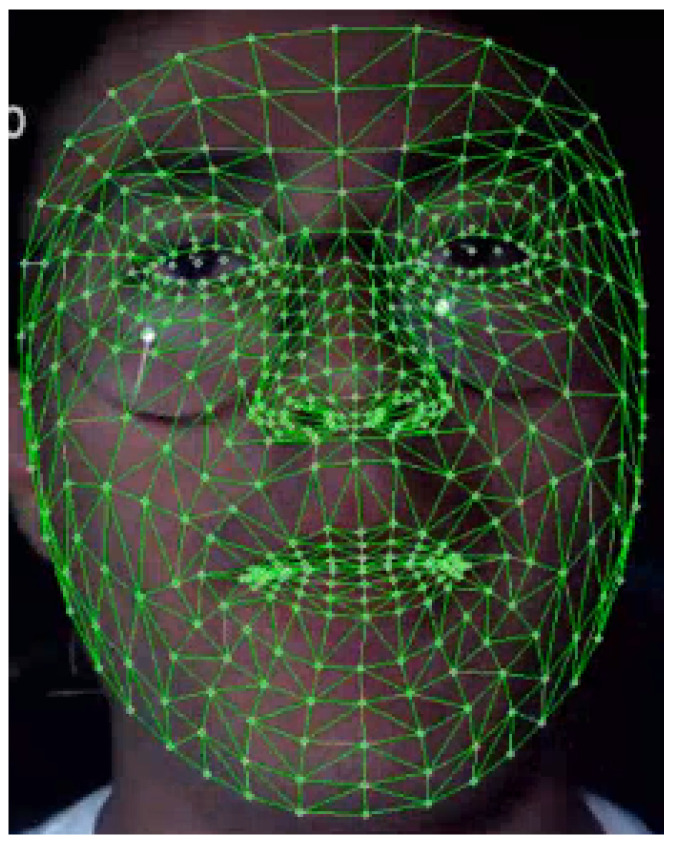
Face detection and dense facial landmark mesh (468 landmarks), shown in green.

This system requires the driver’s face as input because it uses MediaPipe Face Mesh framework to generate a facial mask based on 468 landmarks that identify different facial regions, as shown in Figure 2; in this case, the focus is on the eyes. The eye aspect ratio (EAR) is a scalar value that reflects the openness of the eyes; the value increases when the eyes are open and decreases when the eyes are being closed. For a more accurate measurement, the EAR used to determine the drowsiness is the EAR average of both eyes.

**Figure 3:**
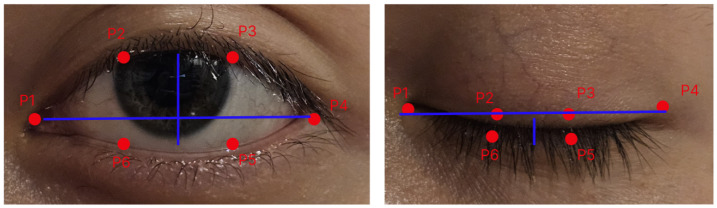
EAR parameter.

To obtain the EAR value, six specific landmarks around each eye have to be used. The landmarks needed are two horizontal points at the corners of the eyes and four vertical points positioned on the upper and lower eyelids. The locations of the landmarks are shown in Figure 3.

The EAR is calculated using the Euclidean distance between each pair of landmarks. The formula to obtain the value is [12]

$$EAR=\frac{∥p_{2}-p_{6}∥+∥p_{3}-p_{5}∥}{2\times ∥p_{1}-p_{4}∥}$$

where the points $p_{1}$ and $p_{4}$ represent the horizontal corners of the eyes. The pair of points $p_{2}$, $p_{6}$ represents the upper eyelids, and $p_{3}$, $p_{5}$ represent the lower eyelids. The landmarks used for each point are 33, 160, 158, 133, 153 and 144, based on Mediapipe Face Mesh [13].

### 2.5. System for Drowsiness Detection Based on Mouth Aspect Ratio (MAR)

This system also uses face detection, following a methodology analogous to the EAR computation, to determine an additional parameter focused on the mouth region. The Mouth Aspect Ratio (MAR) is a scalar value that describes the degree of mouth opening and is commonly used to detect facial actions such as yawning, speaking, and laughing. In this case, the focus is on yawning as an early indicator of drowsiness.

**Figure 4:**
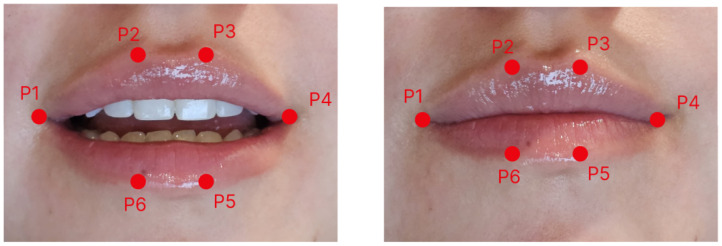
MAR landmarks.

To obtain the MAR value, six specific landmarks around the mouth are needed. These landmarks include two horizontal points at the corners of the mouth and four vertical points located on the upper and lower lips. The location of the landmarks is shown on Figure 4.

However, the MAR is inherently sensitive to partial yawns and other mouth-related actions such as speech or laughter. This limitation motivates the use of temporal analysis and decision smoothing, rather than relying solely on single-frame MAR values, particularly in real-world driving scenarios.

The MAR is calculated using the Euclidean distance between each pair of landmarks. The formula to obtain the value is [14]

$$MAR=\frac{∥p_{2}-p_{6}∥+∥p_{3}-p_{5}∥}{2\times ∥p_{1}-p_{4}∥}$$

where the points $p_{1}$ and $p_{4}$ represent the horizontal corners of the mouth. The pairs of points $p_{2}$, $p_{6}$ and $p_{3}$, $p_{5}$ represent the upper and lower inner lips, respectively. The landmarks used for each point are 61, 65, 63, 67, 64 and 66, based on MediaPipe Face Mesh.

### 2.6. System for Distraction Detection Based on Face Position

**Figure 5:**
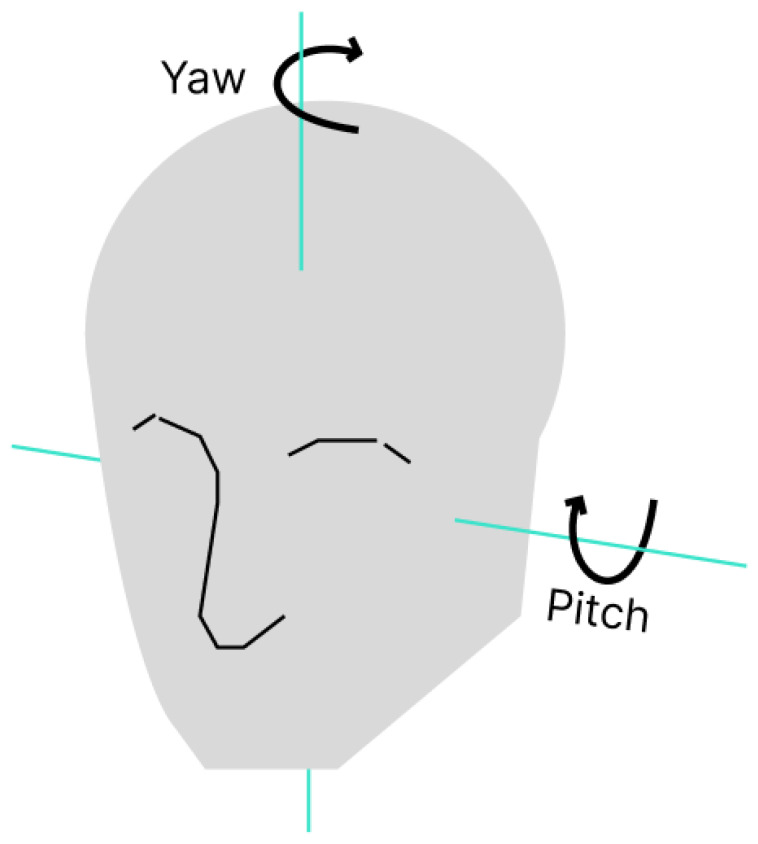
Axes of head rotation.

This system relies entirely on facial landmark detection to compute two parameters related to driver distraction. The first parameter is the position of the irises, which indicates whether the driver’s gaze is directed toward the road or diverted toward other objects. Gaze diversion is one of the most common causes of traffic accidents. The second parameter is the inclination of the head, mainly along two axes, known as pitch and yaw [15]; both are visible in Figure 5. These movements are indicators of the orientation of the head and can indicate whether the driver’s gaze is centered on the road or turned for a prolonged period of time, which may indicate distraction. Both parameters must be calibrated to avoid false positives due to the natural movement of the eyes and the head when the driver check mirrors, reads traffic signs or participates in observer elements that are part of a normal driving behavior.

The distraction recognition zones (left, right, upward, and downward) were defined conservatively and evaluated in conjunction with temporal constraints to avoid penalizing brief, normal driving behaviors such as mirror checking or traffic-sign scanning. A distraction event is only registered when gaze deviation or head orientation persists beyond a predefined duration, improving efficiency while preserving sensitivity to sustained attentional shifts.

### 2.7. Python Script

**Figure 6:**
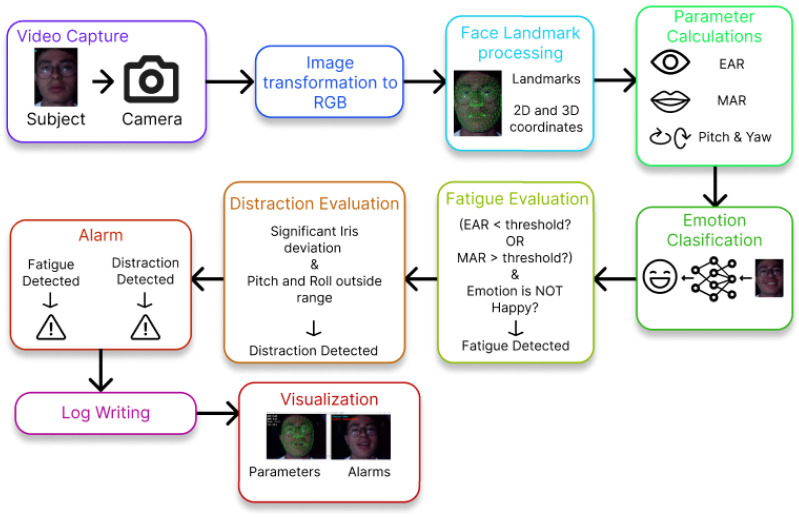
Schematic diagram of the Python-based workflow used for real-time driver monitoring, from video capture and facial landmark processing to fatigue, distraction, and emotion analysis.

The functionalities previously described were combined in a single Python 3.10 script. The main objective is to detect drowsiness and distraction, while the emotions are recognized and recorded. Figure 6 is a flow diagram of the operation of said script.

All the systems described previously include filters to improve their effectiveness. Most of these filters apply a threshold and a grace period before detecting drowsiness or distraction in order to reduce false positives given to certain movements or natural actions in normal driving behavior. Also, the parameters EAR and MAR for drowsiness detection have more importance than distraction detection when both parameters are detected, due the critical nature of this.

Frame-by-frame measurements are not evaluated in isolation. Instead, the system requires consistent facial landmark-based estimates across consecutive frames before updating the driver state. This temporal consistency reduces sensitivity to sporadic landmark jitter or brief focus variations while preserving meaningful transient cues that persist over time.

Additionally, temporal constraints were applied to mitigate false positives caused by sudden illumination changes, such as passing vehicle headlights or rapid lighting transitions. An alert is triggered only when threshold violations persist for a predefined duration, preventing spurious detections due to transient brightness fluctuations.

Fixed thresholds were employed to preserve real-time performance, computational efficiency, and reproducibility across subjects and devices. To reduce false alarms, these thresholds were combined with temporal constraints (grace periods) and prioritization logic, for which fatigue detection takes precedence over distraction detection when both conditions are present. Nonetheless, we acknowledge that adaptive thresholds calibrated per subject and environmental context represent a relevant improvement for large-scale deployment.

### 2.8. Tests

In addition to the algorithmic considerations, several parameters were defined to ensure the proper functionality and stability of the proposed system. These parameters are mainly related to the experimental environment in which the tests were conducted, as well as to the hardware and software specifications required for reliable execution. The experimental platform is based on an Intel Core i7-12700 processor (base frequency: 3.4 GHz) and an NVIDIA RTX 3070 GPU, which was used to train the convolutional neural network using CUDA 11.8. The system was implemented in Python 3.10 and relies on the MediaPipe framework (v0.10.21) for facial landmark detection. Additional core libraries include OpenCV (v4.5.5) for image processing, PyTorch (v2.7.1 + CUDA 11.8) for deep learning inference, and Pygame (v2.6.1) for audio-based alert management.

As far as the environment, the tests were conducted inside a car, where the illumination and the distance of the participants from the camera were similar, to mainly probe the effectiveness of the system on the environment it was made for. Some other tests were carried out in an outside setting, with the illumination changing between tests, and the posture of the participants were really different, according to each person.

Low-light scenarios were intentionally included to approximate realistic night-driving conditions, under which fatigue-related events are more likely to occur. This design choice allowed us to evaluate the stability of facial landmark detection and the reliability of the alert mechanisms under challenging illumination conditions commonly encountered in real-world driving.

## 3. Results

### 3.1. System Evaluation Using Images

**Figure 7:**
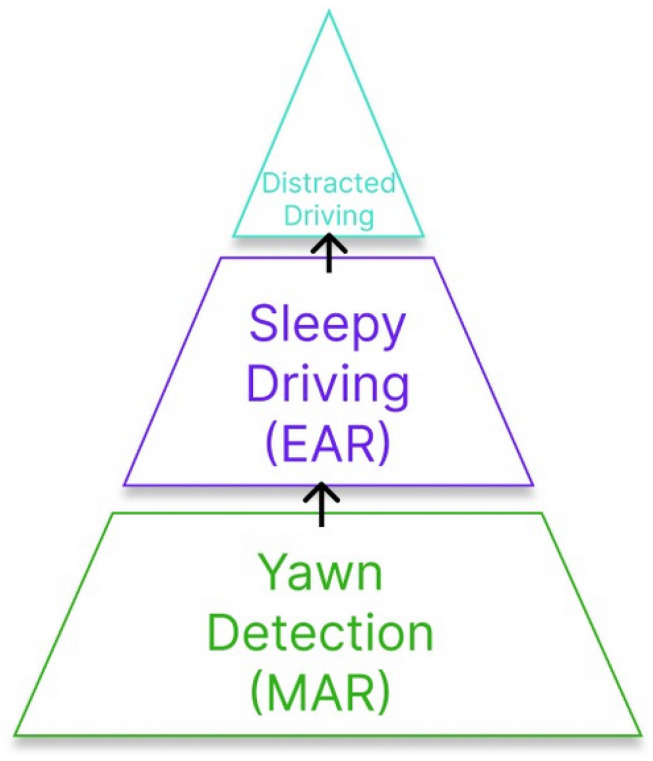
Hierarchy of the system evaluation.

Before the implementation, a general evaluation was conducted using the data set “Driver Inattention Detection Dataset” [11] with the classes distracted driving, sleepy driving and yawning. Each category helped test the accuracy of the distraction detection, the EAR and the MAR for drowsiness detection. Every picture went through the system, with yawn detection evaluated first and sleepy driving next, and at the end, distracted driving was evaluated. With this order, false positives are avoided in pictures. The hierarchy is shown in Figure 7.

**Figure 8:**
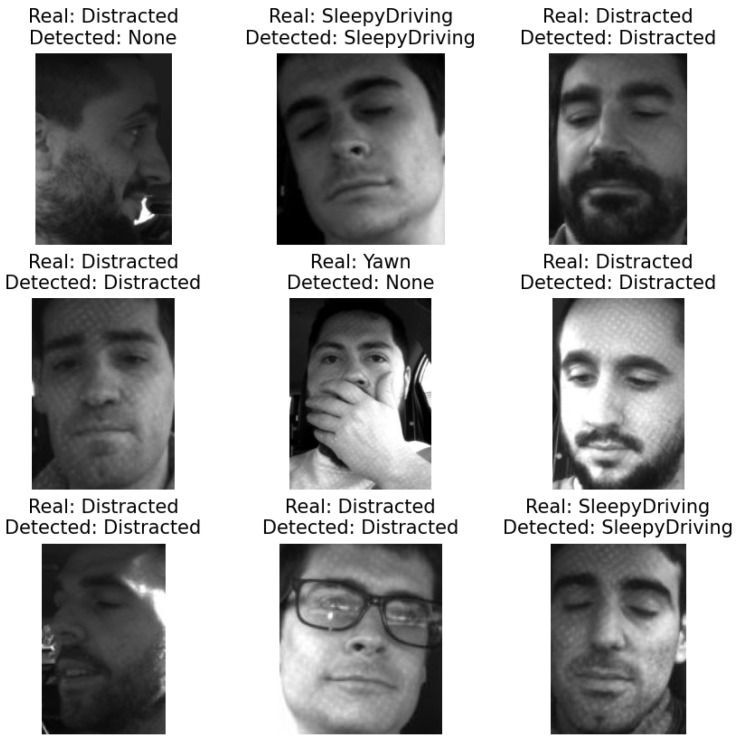
Example of the system’s evaluation using the inattention dataset [11].

After the evaluation of the systems, the following results were obtained. For the detection of distracted driving, the system achieved 84.71% precision, while the sleepy driving detection associated with the EAR achieved 99.9% precision, and finally, yawns related to the MAR achieved 36.84% precision. The low precision in yawn detection was due to some images in which the person’s face was partially covered. Also, other problems were found with the head position of some people, provoking errors with face detection and landmarks. In Figure 8, some tests are shown, in which every example indicates the real class of the image and the output of the system.

Given the results of this test, the system was finally implemented on a real situation inside a car.

### 3.2. Examples of Operation on Real Time

**Figure 9:**
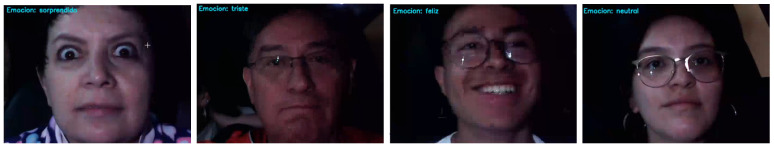
Emotion identification results (surprised, sad, happy and neutral).

The system was able to detect emotions, as seen in Figure 9. One of the screen recordings was generated via the Python script expressed in the upper left corner the state of emotion of the subject. The system could identify five states: surprise, anger/disgust, sadness, happiness and neutrality.

**Figure 10:**
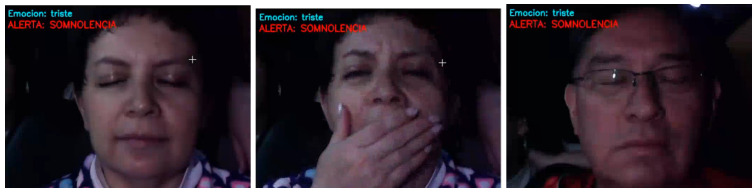
Fatigue alarm.

**Figure 11:**
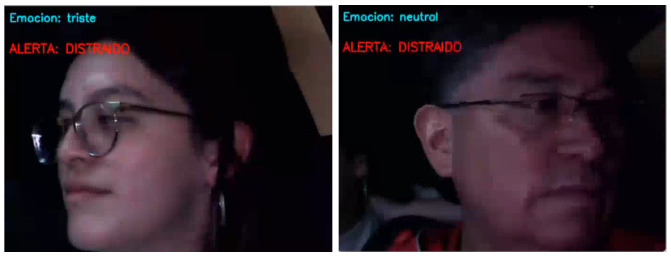
Distraction alarm.

On the other hand, the alerts for both drowsiness and distraction also took place effectively, as can be seen in Figure 10 and Figure 11. The system could detect fatigue in cases where the subject presented only closed eyes and also in cases where there was yawning (even in cases were the yawning was obscured by hand position).

In the case of a distraction alert, the system was able to identify the subject’s head movements and determine whether the gaze was directed to the right, left, up, down, or away from the road. For both distraction and drowsiness alerts, a visual sign in bright red appeared at the upper left corner of the screen recording. The alerts also consisted of a sound being played by the PC that was meant to signal the subject to pay attention or wake up.

### 3.3. Detection Accuracy Results for Driver State Monitoring Tasks

Table 2 displays the unprocessed results from real-time detection experiments involving 27 individuals, targeting six distinct driving behaviors. These include yawning, eye closure (EAR < 0.23), proper driving posture (facing forward), and various distractions such as glancing right, left, upward, or downward (e.g., phone usage).

From the 27 evaluations per behavior, the system accurately identified 23 yawning events, 24 instances of closed eyes, and 20 occurrences of standard forward-facing posture, with a perfect detection rate (27/27) for all distraction-related behaviors.

**Figure 12:**
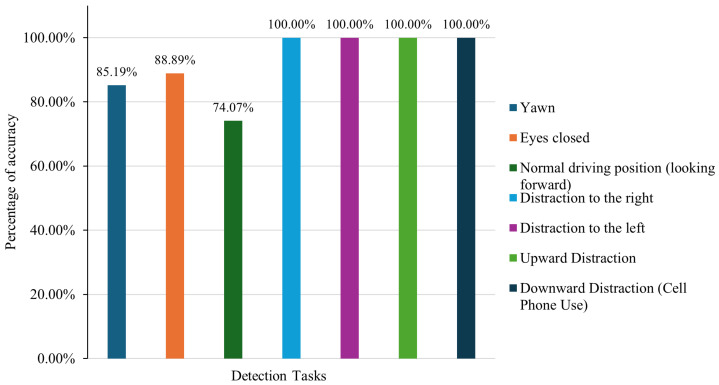
Performance evaluation of driver state monitoring system across detection scenarios.

The bar chart shown in Figure 12 presents the detection accuracy achieved by a driver monitoring system across several tasks aimed at identifying drowsiness and distraction in drivers. In general, the findings underscore the system’s high efficacy in recognizing distraction-related behaviors. All cases involving gaze deviation—whether directed to the right, left, upward, or downward—achieved a perfect detection rate of 100%, demonstrating the reliability of the algorithm’s yaw, pitch, and iris deviation computations.

Nevertheless, the category labeled “Normal driving position (looking forward)” showed the lowest detection rate (20/27), recording an accuracy of just 74.07%, the lowest among all detection tasks. This outcome corresponds with field observations in which some participants were incorrectly classified as distracted despite facing forward in a proper driving posture. This discrepancy is primarily due to the sensitivity of the pitch and yaw thresholds. Variations in distance from the camera or differences in facial geometry may have caused the yaw and pitch estimations to fall outside acceptable ranges, resulting in false identifications.

Eye closure detection yielded relatively strong results with an 88.89% accuracy rate. However, misclassifications were observed among participants with smaller eyes, for whom the Eye Aspect Ratio (EAR) remained below the defined threshold even when the eyes were open. This led the system to mistakenly interpret their state as drowsy. These errors highlight the need to implement facial normalization techniques or adaptive thresholding mechanisms in future system designs.

To mitigate this limitation, a subject-specific baseline calibration strategy is proposed for future system iterations. In this approach, the Eye Aspect Ratio (EAR) would be estimated during an initial alert driving period to define an individualized open-eye reference. The drowsiness detection threshold would then be computed relative to this baseline, rather than using a fixed global value, thereby reducing false positives for drivers with naturally smaller palpebral fissures.

Yawning was detected with an accuracy of 85.19%, primarily based on mouth opening quantified through the Mouth Aspect Ratio (MAR). However, during artificially induced yawns, participants often did not fully open their mouths, resulting in only minor MAR fluctuations. This indicates that yawning is difficult to simulate reliably and suggests that temporal sequence analysis may be more effective than relying solely on single-frame detection. To address this limitation, future versions of the system will incorporate temporal features such as MAR trends over time, the duration for which MAR values remain above a predefined threshold, and yawn event counting. These sequence-based descriptors are expected to improve robustness by distinguishing genuine yawning from transient or partial mouth openings.

On the other hand, all types of distraction, including changes in gaze direction and mobile phone use, were accurately identified in every instance. This confirms the effectiveness of combining head pose estimation with iris tracking as a robust method for detecting attentional shifts.

It should be noted that the reported 100% accuracy for distraction detection corresponds to the specific scenarios evaluated in this study, which primarily involved deliberate and sustained gaze deviations (left, right, upward, and downward), including controlled mobile phone usage. More complex and subtle distraction behaviors, such as eating, gesturing while conversing, or brief secondary task interactions, were not explicitly tested. Therefore, the reported performance reflects effectiveness within the defined experimental scope, rather than exhaustive real-world distraction coverage.

Finally, it is worth noting that improved lighting enhanced facial landmark detection and reduced inaccuracies in EAR, MAR, and head pose measurements, contributing to better overall system reliability. This observation highlights the importance of illumination-aware pre-processing techniques, such as adaptive exposure control or image normalization, as practical enhancements for improving system robustness under varying lighting conditions.

In summary, the system shows an excellent capability of identifying distraction and considerable effectiveness in detecting signs of drowsiness. The current limitations are mostly due to variability in facial characteristics, lighting conditions, and positioning relative to the camera. A practical enhancement to further address illumination variability is the incorporation of illumination-aware pre-processing techniques, such as adaptive histogram equalization or exposure normalization, prior to facial landmark extraction. These findings will support the development of improved calibration strategies for better real-world effectiveness.

### 3.4. Statistical Analysis of Eye Aspect Ratio Algorithm (EAR) Measurements

**Figure 13:**
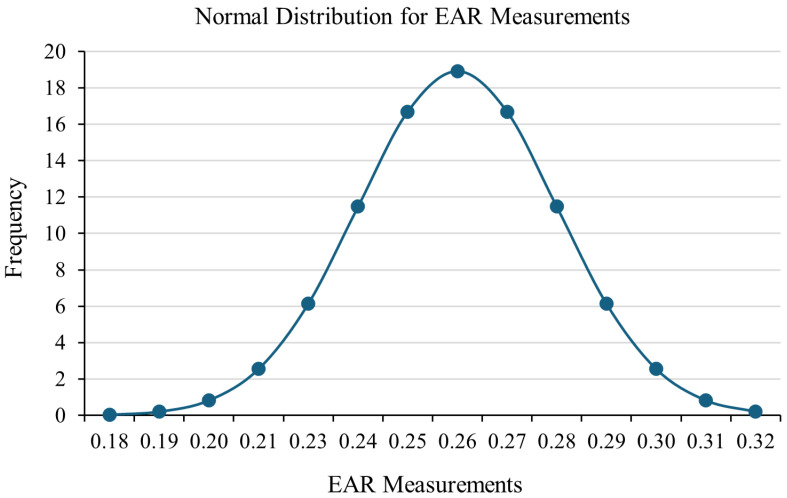
Normal distribution for EAR measurements from 27 participants.

To assess whether the selected Eye Aspect Ratio (EAR) threshold is suitable for detecting fatigue, a statistical analysis was conducted using EAR values from 27 participants who were fully alert. As shown in Figure 13, the data followed a normal distribution with a mean EAR of 0.2570 and a standard deviation of 0.02109. This indicates that, under normal conditions, EAR values are generally stable between individuals, even though some differences due to facial structure are expected.

In this research, the system did not use individual calibration. Instead, it applied a fixed EAR threshold of 0.23 to detect signs of drowsiness. This value was chosen based on earlier studies, especially by Yuen et al. [16], who found 0.225 to be a good threshold for detecting fatigue under different lighting and age conditions, with very high accuracy. The slightly higher threshold of 0.23 was selected to improve the system’s reliability in detecting longer eye closures while reducing errors caused by normal blinking or facial movements.

The analysis supports this threshold choice. Since 0.23 is about 1.3 standard deviations below the average EAR, it allows the system to clearly tell the difference between open and closed eyes for most users. However, because people’s EAR values can vary, using personalized thresholds could help improve accuracy in future versions of the system, especially for users whose normal EAR is far from the average.

In addition to population-level thresholding, a practical enhancement is adaptive thresholding based on each driver’s baseline EAR during alert conditions. This approach is expected to reduce false positives for users with naturally lower EAR values (e.g., smaller palpebral fissures).

While fixed thresholds were adopted in this work, their selection is supported by both the prior literature and the population-level statistical distribution observed in the collected data. Adaptive thresholding strategies could further enhance robustness without compromising the statistical validity of the current approach.

### 3.5. Performance Evaluation of Emotion Detection

**Figure 14:**
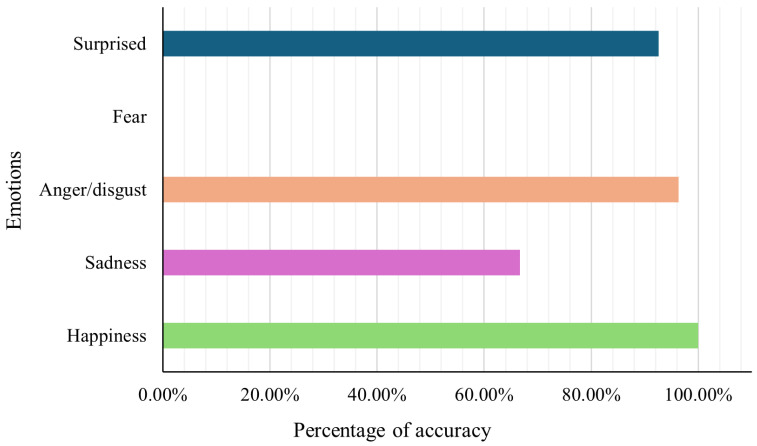
Accuracy of emotion detection across tested participants.

Figure 14 shows how well the emotion detection system worked in both real and simulated driving scenarios. The system did especially well at recognizing happiness (100%), anger/disgust (96.3%), and surprise (92.6%). These emotions tend to show up clearly on people’s faces, which likely helped the system identify them more accurately.

On the other hand, sadness was harder for the system to detect, with an accuracy of just 66.7%. Although sadness is not as expressive as other emotions, it is still important to track because it can affect a driver’s focus and reaction speed. Sani et al. found that sadness lowers a driver’s awareness of their surroundings, which can lead to slower responses and an increased chance of accidents—making it a critical emotion to monitor [17].

Interestingly, the system could not detect fear at all. This seems to be because the training images from the RAF-DB dataset show overly dramatic fear expressions that do not match how people usually look when they are afraid while driving. As a result, the system struggled to recognize fear in more natural, everyday settings. This highlights a need for training data that reflects more realistic emotional expressions. These findings are consistent with a domain-shift effect between laboratory-style datasets and in-vehicle facial behavior. Therefore, while RAF-DB enables lightweight training and broad emotion coverage, future work will evaluate transfer learning strategies (e.g., fine-tuning on driving-oriented data or domain-adaptive augmentation) to better capture subtle and context-dependent expressions observed during real driving.

Anger and disgust were combined into one category since drivers often showed them with similar facial features. This decision is backed by research showing that both emotions cause similar physical and behavioral reactions, like increased arousal and aggressive driving habits. Sani et al. also point out that anger can lead to risky actions, such as speeding and following too closely, which significantly raise the chances of a crash [17].

Overall, despite some limitations, the system shows strong potential for monitoring drivers’ emotions in real time. It can reliably detect emotions like anger, surprise, and happiness, which are known to affect attention and decision-making. This makes it a promising tool for helping prevent accidents by identifying emotional states that could put drivers at risk.

## 4. Discussion

The idea of projects designed to prevent vehicular accidents caused by driver’s fatigue is one that has been implemented in the field of artificial vision before. The work by [18] used OpenCV to monitor the blinking frequency of the driver; even if the drowsiness metric is different compared to this work, it remains a constant to check on the subject’s eyes to tell whether they are falling asleep. In [19], a CNN system used artificial vision to determine the level of drowsiness of drivers based on eye closure, blinking frequency and yawning frequency. A future objective could be to add various eye tracking functions to make the identification of a fatigue state more robust; the addition of other metrics like a yawn counter could also be useful to increase the system’s accuracy.

In the initial tests of the system, the Eye Aspect Ratio (EAR) achieved high accuracy, whereas the Mouth Aspect Ratio (MAR) exhibited lower accuracy due to variations in the natural head positions of the participants. To address this limitation, additional landmark-based metrics can be incorporated. As reported in [20], MAR achieved an initial accuracy of 58%, which was subsequently improved by complementing it with Face Length (FL) and Face Width Balance (FWB) features, all of which were combined using artificial neural networks (ANNs). The calculation of all parameters allows for a proper detection of the MAR when people adopt different postures, obtaining accuracy of 72%.

The use of constant parameters for the EAR, the MAR, and head-pose estimation reflects a trade-off between system simplicity and adaptability. Fixed thresholds facilitate low-latency operation and reproducibility, which are critical for real-time driver monitoring systems. However, adaptive and personalized calibration strategies could better account for inter-subject variability and changing environmental conditions, and they will be explored in future iterations of the system.

A key limitation for emotion recognition is the mismatch between generic facial expression datasets and naturalistic driving conditions. In particular, some categories (e.g., fear) may be underrepresented or expressed more subtly in real driving, which can reduce detection rates when models are trained on exaggerated portrayals. Future iterations will explore domain adaptation through fine-tuning on driving-specific emotion data (when available) and the incorporation of temporal modeling to better capture subtle facial dynamics under real-world constraints.

In order to improve distraction detection, not only head movement is considered, but gaze estimation is also taken into account. As reported in [21], relying solely on head pose estimation results in an average error of approximately 6°, which can limit its reliability in real-world applications. For this reason, detection accuracy can be significantly improved by combining head pose and gaze estimation, as these parameters are correlated and complementary. This approach is consistent with the proposed system, in which both head pose and gaze information are required to reliably detect driver distraction.

Head pose estimation alone still yielded an average error of around 6°, which limits its reliability for real-world applications. The authors, therefore, suggest that combining head pose with gaze estimation can significantly enhance accuracy since both parameters are correlated and provide complementary information. This perspective aligns with the present work, where gaze is integrated as an additional feature to strengthen the robustness of distraction detection.

The methodology developed in this work can also be compared with other approaches based on fatigue identification models In [3], the authors developed a fatigue identification system based on SVMs (state vector machines), which yielded 0.95 accuracy in the results. Perhaps the system developed in this work could benefit from having a custom-made model for fatigue detection; a similar one could be implemented for the case of a distracted state, too. In the work of [22], the trained model for fatigue recognition is YOLOv8, the tests produced a recognition precision of 0.91. A long short-term memory network and a long recurrent convolutional network were developed to detect driver activity in [23], which yielded almost 0.98 accuracy. There seems to be the case in which custom-built and trained models produce better results than widely available, open-source ones like the ones used for this article.

A direct quantitative comparison with state-of-the-art methods under identical experimental conditions was not conducted in this study. This was primarily due to differences in the task scope, datasets, and evaluation objectives. Most related works focus on single-task detection (e.g., fatigue or distraction alone) using task-specific models and curated datasets, whereas the proposed system integrates fatigue, distraction, and emotion recognition into a unified, real-time framework. Therefore, the contribution of this work is not centered on outperforming specialized single-task architectures in isolated metrics but on demonstrating the feasibility and effectiveness of a lightweight, multi-task driver monitoring system capable of operating in real time using non-invasive visual cues. Future work may include benchmarking against selected baseline methods using a unified dataset to enable more direct quantitative comparisons.

On the other hand, the work presented in [5] employs a more comprehensive system than the one proposed in this study, as it incorporates biometric measurements to further analyze the subject’s state. Although this fatigue detection approach is relatively intrusive, the integration of biometric sensing represents an interesting direction for future extensions of the present work. Furthermore, a CNN-based system was proposed in [24] to detect driver activity using driving-related signals such as acceleration, throttle position, and vehicle speed. Studies such as these raise the question of whether additional information sources beyond computer-vision-based methods could be leveraged to identify the driver’s fatigue level and emotional state.

A point of contention within the methodology of this work is the fact that it could have used more structured tests to verify its effectiveness against near-real-life scenarios. In [25], the tests were conducted on subjects who were actually driving for considerable periods of time, therefore making the collected data a lot more reliable.

Another limitation of the current validation is that the experiments were conducted in short-duration sessions under simulated or static driving conditions. While these tests are sufficient to evaluate real-time detection accuracy, system responsiveness, and robustness to transient behaviors, they do not capture fatigue accumulation or evolving distraction patterns that occur during prolonged driving. Long-term evaluations in real driving scenarios, including continuous operation over extended periods, are therefore a necessary next step to assess system endurance, temporal stability, and practical reliability under realistic fatigue progression.

The system can be further developed in future work. It could be beneficial to make the system personalized for each subject so as to account for the variances in the EAR parameter. Model training could also be applied in the case of facial landmarks’ detection to strengthen the effectiveness of fatigue and distraction identification.

## 5. Conclusions

To create a robust system able to detect negative behaviors while driving, different systems are needed to secure the proper detection of drowsiness, distraction and negative emotions. All systems must include filters and thresholds according to the values obtained from previous tests to avoid false detections and ensure that all the information obtained is real.

The use of CNNs and pre-trained models, alongside Python OpenCV, allowed the development and implementation of a state detection system for drivers.

The system effectively combined facial landmark detection with deep learning techniques to monitor driver states in real time. By leveraging MediaPipe’s 468 landmarks, it accurately tracked eye and mouth movements, as well as head pose and gaze direction. This enabled the reliable identification of drowsiness, distraction, and emotional changes during driving scenarios. The implementation proved technically feasible and efficient for real-time applications.

Distraction detection achieved the highest performance, with 100% accuracy across all tested gaze deviation scenarios. This confirmed the robustness of using head pose angles and iris positioning to recognize attentional shifts. In contrast, the detection of proper forward-facing posture showed slightly lower performance, mainly due to sensitivity in pitch and yaw thresholds. This highlighted the importance of accounting for natural facial variability during calibration.

Drowsiness detection using the Eye Aspect Ratio (EAR) and the Mouth Aspect Ratio (MAR) showed solid results, with detection rates of 88.89% and 85.19%, respectively. However, certain limitations arose from differences in facial geometry and participants’ simulation of yawning. The EAR threshold of 0.23, chosen based on prior research, aligned well with the statistical distribution of participants’ data, supporting its use without prior individual calibration.

The emotion recognition system based on MobileNetV2 achieved high accuracy for expressions like happiness, anger/disgust, and surprise. Yet, it struggled with more subtle or less visually distinct emotions such as sadness and fear, which were either inconsistently detected or not detected at all. This issue was attributed to discrepancies between real-world expressions and those found in the training dataset (RAF-DB), particularly for fear.

By integrating emotional context into the fatigue and distraction detection pipeline, the system reduced false positives caused by expressive facial movements like smiling. This strategy allowed for more nuanced decision-making in behavioral classification. Overall, the system demonstrated a practical and scalable approach to enhancing in-vehicle driver safety through the non-invasive, real-time monitoring of visual cues.

## Figures and Tables

**Table 1:** Summary of participant characteristics.

Characteristic	Value
Number of participants	27
Age range	24–50 years
Gender distribution	10 female/17 male
Driving experience	Licensed drivers (>3 years)
Nationality	Ecuadorian
Cultural background	Ecuadorian highlands (Sierra region)

**Table 2:** Detection results for specific driver actions using the monitoring system.

Accuracy/Action	Yawn	Eyes Closed	Normal Driving Position	Distraction Right	Distraction Left	Upward Distraction	Downward Distraction
NO	4	3	7	0	0	0	0
YES	23	24	20	27	27	27	27

## Data Availability

The RAF-DB dataset used for emotion recognition training is publicly available at https://www.kaggle.com/datasets/shuvoalok/raf-db-dataset. URL (accessed on 15 July 2025). The Driver Inattention Detection Dataset used for system evaluation is available at https://www.kaggle.com/datasets/zeyad1mashhour/driver-inattention-detection-dataset. URL (accessed on 15 July 2025). The real-world dataset used for live system validation consists of private data collected from volunteer participants with informed consent and is not publicly available due to privacy and ethical restrictions.
